# “Can You Deny Her That?” Processes of Governmentality and Socialization of Parents in Elite Women’s Gymnastics

**DOI:** 10.3389/fpsyg.2022.829352

**Published:** 2022-05-31

**Authors:** Froukje Smits, Frank Jacobs, Annelies Knoppers

**Affiliations:** ^1^Institute Social Work, HU University of Applied Sciences Utrecht, Utrecht, Netherlands; ^2^Department of Health, Nutrition and Sports, The Hague University of Applied Sciences, The Hague, Netherlands; ^3^Utrecht School of Governance, Utrecht University, Utrecht, Netherlands

**Keywords:** gymnastics, parents, socialization, governmentality, elite

## Abstract

Abusive practices in elite women’s artistic gymnastics (WAG) have been the focus of discussions about how to eliminate or reduce them. Both coaches and parents have been named as key actors in bringing about change. Our focus is on parents and their ability to safeguard their daughters in WAG. Parents are not independent actors, however, but are part of a larger web consisting of an entanglement of emotions and technologies and rationalities used by staff, other parents, and athletes, bounded by skill development plans and by coaching expertise and authority. This entanglement may limit the ability of parents to bring about change. We draw on a Deleuzian notion of assemblage, Foucauldian concepts of discourse and governmentality and Ahmed’s assertion about the entanglement of discourses and emotions to explore how parents are disciplined into accepting dominant discursive practices of sport clubs for elite athletes. The data were drawn from a project called the Parental Awareness Program (PAP) that was designed to make parents aware of practices in competitive WAG that may not be in their child’s best interest. Participants were parents of young gymnasts who had been identified as “talented” and who were members of an elite gymnastics club. The data analysis was based on focus group discussions with a total of 22 parents and semi-structured interviews with 8 parents. The results suggested that although parents problematized many practices during PAP, processes of governmentality involving an assemblage of discourses about coaching expertise, families, talent, enjoyment, long term skill development plans and its associated time demands, together ensured parental consent for dominant practices. The data suggested that a reduction of abusive practices lies in part in critical examinations of skill development plans that are presented as regimes of truth and are kept in place by emotions and the authority accorded to coaching expertise. These processes curtail parents in their ability to safeguard what is in the best pedagogical interest of their daughter.

## Introduction

Parents have always been part of youth sport, albeit serving in different capacities ranging from being chauffeurs, uniform washers, coaches, and supporters to working in the cafeteria and marking the fields. Their importance to youth sport should not be underestimated as they enable their children to participate in sport (Knight et al., 2020). The exact nature of parental involvement and influence appear to be shaped by both individual and contextual factors that include the context itself, the actions of other parents and coaches, their own experiences as athletes and as sport parents and their expectations for their child (Knight et al., 2016; Knight, 2019).

The dominant stereotype of parents of talented youth sport athletes, however, is that they are helicopter parents with irrational demands of coaches (Knight et al., 2017). It is not surprising then, that empirical research on parents and their specific contributions is largely limited to how parents can be involved in a positive way so that they do not put pressure on their children to produce outstanding performances or on coaches to produce champion athletes. The focus of a great deal of research in the last 10 years has been on how parents can be discouraged from trying to control their child’s sporting experiences (Kerr and Stirling, 2012; Booth et al., 2014; Clarke et al., 2016).

Parents may, however, also become socialized or disciplined into a normalized culture of a sport and in so doing, may tacitly accept or ignore training practices, organizational factors/routines and emotional, physical, and sexual abusive behaviors and interactions with coaches that place a child at risk (Grenfell and Reinhart, 2003; Holt et al., 2008; Dorsch et al., 2009; Stirling, 2011; Kerr and Stirling, 2012; Holt and Knight, 2014; Stirling and Kerr, 2014). Parents have also realized that if they question coaches they may jeopardize the coach-athlete relationship (Kerr and Stirling, 2012; Smits et al., 2017). As a result, parents may often be silent bystanders in their child’s experiences in sport instead of resisting practices that may not be in their child’s best interest. Knight et al. (2020) argued that in elite sport where:

“winning is centralized and coaches are dominant, parents can find themselves trying to walk a tightrope between being there and available for their children, commenting on practices that they deem inappropriate, and not acting in a manner that may upset a coach and could subsequently impact on their child’s chances of success in sport.” (p. 307)

Relatively few scholars have looked at processes of parental socialization within the context of elite sport. An exception is work by McMahon et al. (2018). They examined parental socialization of elite swimmers and gymnastics. They found parents molded or shaped their behavior to be congruent with cultural narratives about what is needed for performance and perfections in this sport. The socialization processes of parents of elite athletes may mean they do not understand or see how the practices that comprise this training may not always contribute to the positive social development of the child (Lang, 2010a,b; Smits et al., 2017). Smits et al. (2017) found that parents of retired and current elite gymnasts accepted abusive practices because they thought that such practices were normal, that is, specific to elite gymnastics. Most research about parents accepting abusive practices as normal coaching behavior was, however, conducted before media and court cases worldwide drew attention to abusive practices in elite women’s gymnastics. Our project explored current thoughts, attitudes and coping strategies of parents in navigating dominant practices in elite gymnastics. The project was situated in a setting and time in which abusive practices in women’s gymnastics were constantly in the news, had become public knowledge and were also the subject of a parliamentary debate.

The revelations about coaches abusing youth sport athletes such as in gymnastics, have not only been condemned but have also created expectations that parents engage in safeguarding their children from forms of abuse that may occur during their sport participation (Knight et al., 2016, 2020; Harwood et al., 2019). Various scholars have suggested that this socialization of parents into the norms of elite sport culture might be countered by educating them about the forms abuse can take in sport (Mountjoy et al., 2016; McMahon et al., 2018). McMahon et al. (2018) for example, used narrative pedagogy in an education program geared toward parents of elite swimmers and gymnasts. The purpose of the program was to make parents aware of situations where abusive practices can occur. Parents participating in that study agreed the described hypothetical coaching practices presented in the narratives were unacceptable. They also, however, suggested that perhaps such coaching practices could be necessary to help a child improve their performance. Specifically, they rationalized and normalized these abusive behaviors by coaches and constructed the coach as knowing what the best and necessary practices were to produce elite performance. This acceptance of these ways of coaching may make parents unable to challenge practices that might not be in the best interest of their children. McMahon et al. (2018) used narratives about hypothetical situations and athletes, however. These parents might have reacted differently if the narratives had described their own daughters participating in elite gymnastics. Research is needed that explores how parents of elite athletes such as those in gymnastics, currently conceive of themselves as protecting their child in their participation in the sport and how, if at all, parents now problematize practices and the techniques or strategies employed by clubs.

Parents are not independent actors, however, but are part of a larger web consisting of elements such as affect, coaches, sport culture, other parents, and athletes that shape parental identities of potentially outstanding gymnasts (Dorsch et al., 2009, 2015, 2016; McMahon et al., 2018; Smits et al., 2020). These constructions can function as a mechanism of socialization that exerts control with the aim of obtaining parental consent to norms about what is accepted and needed to “develop the good gymnast” and to be the appropriate, compliant or “good parent.” Relatively little scholarly attention has been paid to the multitude of elements that constitute processes of socialization that could make parents complicit in accepting and normalizing current practices in elite gymnastics and/or resisting such practices. In the current study we explore how parents thought about the processes of the development of their daughters as elite gymnasts and their rationale for their thoughts and how, if at all, they resisted what they saw as problematic practices.

### Purpose of the Study and Research Question

The focus of our study was on how parents of talented young gymnasts navigated the practices, regulations and other elements that shaped their daughter’s participation in elite gymnastics. The research question was: how are parents of elite young gymnasts governed into acquiescence of dominant club practices? Specifically, which elements comprise an assemblage of practices, regulations, knowledges and other forces that may interact to constitute parents of elite young gymnasts and which processes of governmentality socialize parents into acquiescence and acceptance of current practices?

## Theoretical Framework

Our study is based on comments made by parents during a Parental Awareness Program (PAP). The goal of the program was to enable parents to counter the strategies used by the club/staff and by their daughters to shape them into being proper gymnastic parents. We uncovered several elements that together comprised an assemblage that was subjected to processes of governmentality that socialized parents into acquiescence and acceptance of current practices. We foreground relations of power and governmentalities as they are constituted in regimes of practice or assemblages.

We used a lens based on Buchanan’s (2015) conceptualization of assemblage, Foucault’s (Foucault, 1974, 1991, 2003) notion of discourses and governmentality, and Ahmed’s (2010) contention about the entanglement of discourses and emotions to explore how parents were disciplined or governed into accepting current practices in elite gymnastics. Buchanan (2015) used a Deleuzian notion of assemblage defining it as “the productive intersection of a form of content (actions, bodies and things) and a form of expression (affects, words and ideas)” (p. 390). Specifically, an assemblage in the context of this study can be seen as the assumption that a multitude of practices, regulations, knowledges and other forces interact and construct what it means to be a parent of an elite gymnast (Brown et al., 2012). Assemblages are, however, shaped by discourses and relations of power.

A Foucauldian notion of discourse refers to “what can be said and thought, who can speak, when and with what authority ways of thinking and doing that may become truth or common sense, often known as regimes of truth” (Ball, 1990, p. 2). Power is therefore, not seen as coercive but rather involves steering individuals toward specific ways of thinking and behaving that over time become common sense. Governmentality can be defined as acting on the actions of others or the act of conducting the conduct of others (Foucault, 2003). It describes a form of power that is not exercised through rules, but through ways of expressing “truths” that emerge from discourses using rationalities and ways of doing (technologies). These rationalities and technologies emerge from conversations, traditions, culture, and experts that together encourage individuals to regulate their own behavior in a desired direction (Foucault, 1991, 2003). Governmentality in the specific context of the study refers to the shaping or governance of parental behavior and thinking as they engage with an assemblage that is specific to women’s artistic gymnastics (WAG) (Rose, 2000; Dean, 2010; Buchanan, 2015). An explanation of how this type of non-coercive power circulates in a specific context requires an exploration of the technologies (the doing) and rationalities (the thinking) that are used to socialize or regulate into this acceptance.

Emotions are also a large part of sport, however (Sinden, 2013). An initial quick scan of our data suggested perceptions of enjoyment and other emotions played a large role in the socialization of parents. Ahmed (2010) has argued that in addition to discourses, emotions can also shape human behavior and thinking. Emotion, therefore, can also serve as an element in governmentality (see also Burrows and Wright, 2007). A gymnast may for example, feel joy and share this enjoyment with her parents when she nails a specific move on a balance beam. The difficulty of this move is situated in discourses. Specifically, the gymnast feels joy (emotion) because she properly executed what is known (discourse) as a difficult move. Emotion/affect and the discourse are, therefore, inseparable and cannot be distinguished from each other (Ahmed, 2010).

The use of a governmentality analytic therefore, requires researchers to pay attention to how power is exercised through regimes or assemblages of rationalities (ways of thinking) and technologies (ways of doing) and how these assemblages are rooted in history/culture/traditions of the phenomenon under study. Following Ahmed (2010), we argue that emotions are entangled in each of these dimensions and in the elements comprising the gymnastic assemblage in the specific context under study. Governmentality then, refers to the daily rationalities and technologies that produce an assemblage of elements entangled in an affective web of power that govern/regulate or socialize parents into the proper training of elite gymnasts.

## Context

The talent programs in the selected elite clubs are structured and organized by age and by level of performance. Girls tend to be selected for the talent program when they are young. They begin in the so called “pupil” classes for 7–9-year-olds. “Junior” classes are for those 10–12 years old. The young gymnasts become seniors when they are 13 and (still) judged to be talented. The seniors are further subdivided into three groups based on age: 13–14, 15–17 years and those 18 and older.

In addition to age-related categories there are six different levels of performance in WAG. The highest level is the (inter) national level for talented gymnasts. Gymnasts who participate in the first, second, and third division compete at the national level, those in division four and five compete at the district level while those in division 6 (the lowest level) of performance, compete at the regional level. These differentiations across age and classification are associated with hours of training. Those in the 6th division for example, generally practice 4 h per week while talented gymnasts who are selected as seniors are required to practice up to 19 h per week when they are 13–14 years old; those who are 15–17 years old must practice 23 h while those 18 years and older must train 27 h and more. If a gymnast’s performance level is considered to be subpar, she will be excluded from the talent program; if she wishes, she can continue to participate at a lower level and her hours of practice per week will decrease accordingly. In addition to having fewer hours of practice, the lower division gymnasts are trained by assistant coaches. The elite coaches coach only at the national level. This means that those gymnasts who drop-out of the highest level or are demoted to a lower level, learn less and receive less support from the club. Due to this hierarchy, most young gymnasts (and their parents) want to continue to participate at the highest level to fulfill their desire to develop their talent. We recognize that this development system is contextual. Every country may have its own long term development plan for gymnastics.

## Methodology

### Participants and the Project

The PAP was a collaborative initiative from researchers from a university in a midsized city and the board members of two clubs for elite gymnasts. It was grounded in a national program called “Sport innovator”, sponsored by the Dutch ministry of Health, Welfare and Sport. The boards of the two sport clubs sent a letter of to all parents whose daughters were enrolled in the clubs to invite them to attend/participate in a PAP session. The purpose of each PAP session was to enable parents to share their experiences and to make them aware of possible problematic practices that might be part of their daughter’s involvement in gymnastics. During the PAP sessions the authors shared the results of scholarly research conducted with parents, coaches, club managers and elite gymnasts in the Netherlands. Participants also listened to/read recent (2021) media accounts of abusive coaching behaviors in women’s elite gymnastics in the Netherlands. Subsequently, the participants split into three focus groups in which they reflected on their ideas about and their experiences with their daughter’s participation in elite gymnastics. We used the sessions to collect data for the current research project. The data therefore, were primarily parent-generated.

We asked these parents if we could use their responses for the current research project and if we could record their discussions. They were informed that they could withdraw their consent at any time, that their responses and remarks were confidential and that we would do all we could to keep their responses/comments anonymous. We did this by not referring to them by codes/demographic details in the “Results” section. This not only ensured their anonymity and their concerns about confidentiality and privacy but also prevents readers from projecting their assumptions about gender on the quoted fragments or possibly identifying a parent since the number of elite gymnasts are relatively few in each club. The emphasis of the analysis is on what was said in the focus groups, rather than on the (demographics of) the person who said it. Individuals were not the unit of analysis.

All of the participating parents agreed to participate in the study. The participants in the study were 30 parents whose daughters were involved in one of two elite gymnastics clubs. These young girls (6–12 years old) had been designated as talented by the club and as having potential to compete at the international level.

### Data Collection

We used focus groups to enable and enhance collective interactions among parents. Scholars such as Kitzinger (1994) and Sparkes and Smith (2014) have argued that focus groups stimulate the spontaneous expression of participants as they react to comments made by others. Focus groups are an appropriate method for research situated within a poststructuralist perspective (Madriz, 2000; Freeman, 2006; Smithson, 2008). They are assumed to decrease the impact the researcher may have on a discussion. Focus groups have been used in post-structural research to uncover narratives and the discourses in which they are embedded. A strength of focus group methodology is that it enables research participants to develop ideas collectively, bringing forward their own priorities and perspectives. We did this by using an open structure format in which parents were asked about issues they had encountered and by enabling parental interaction with each other rather than only with the leader. The purpose of the moderator or leader in these types of focus groups is to facilitate the discussion and help identify the issues that emerge. We used a whiteboard to note issues that were mentioned by the group as well as by previous groups. The parents involved in the study were therefore, encouraged to talk to each other rather than react to the leader’s questions. This method tends to foster feelings of solidarity among participants. They can share and/or produce common knowledge about relevant issues in the sport club. As Freeman (2006), who led focus groups of parents about the issue of testing in schools, argued:

*When parents question themselves and each other, when they agree and disagree about what they mean, when they seek approbation from others, when they accept the uncertainty of their interpretations by allowing contradictory claims to enter their discourse, they are engaging substantially, evaluatively, and morally with the topic. The parents do not share stories and then add evaluative commentary; they are thinking and engaging together evaluatively, using the stories as evidence of their thinking” (p. 91).*

We therefore used these focus groups and the interviews to gain a better understanding of the issues these parents considered important with respect to safeguarding their daughter’s participation in elite gymnastics and to uncover how they navigated these issues.

Research and media accounts that described the negative consequences of gymnastic participation for young girls in the Netherlands and the world served as an impetus for dialog among parents. We assumed these accounts gave parents a context and placed them in a critical spectator role. Scholars (Madriz, 2000; Freeman, 2006; Smithson, 2008) have argued that asking focus group participants to respond to a common focal point, in our case, media accounts and research, fosters a collectivistic rather than individual response. Through their engagement with other parents in the focus groups and/or with the results of the focus groups as was the case in the semi-structured interviews, understandings and meanings were constructed that were embedded in discourses about being a good gymnast and a good gymnastic parent.

The purpose of the groups was to enable parents to discuss issues they wanted to raise and to share with others about their daughter’s participation in elite gymnastics. This purpose helped us identify elements comprising the assemblage of elite gymnastics practice. The discussion format in each group was semi structured and centered on three main topics: (1) their responsibilities/role as parents and the dilemmas they faced in supporting their daughter, (2) their requests of and wishes for the ways coaches guided and taught their daughters, and (3) their demands and requests of the board of governance of the club. Each topic was moderated by one of the researchers. The selection and use of these topics were based on our earlier research project that suggested practices in women’s competitive gymnastics do not occur in a vacuum but involve various actors (Knoppers et al., 2015; Jacobs et al., 2017; Smits et al., 2017, 2020). In each discussion group, parents were also asked if they had other issues/questions they wished to discuss. To encourage interaction in the focus groups we used a progressive form of discussion (see also Madriz, 2000; Freeman, 2006; Van Doodewaard et al., 2018). Specifically, each group reacted to main points that emerged from the discussions in the other groups on the same topic.

The first evening, two parents (two mothers of two different gymnasts) attended the PAP session. The second evening was attended by 20 parents (13 mothers and 7 fathers). We asked the parents attending the second session to divide themselves into three similarly sized groups of about 6–7 people each. We assumed that parents would feel most comfortable in discussing issues they faced if parents could form their own groups. The two parents who came as a couple joined the same group. Each group met in a different space and then rotated through the three rooms. Each room had its own discussion leader who focused on one of the topics. Researchers involved with the project served as discussion leaders. The leader in each room used a white board to summarize the elements that parents in the previous group had raised about that topic and what they had said about that topic or specific element. Parents were then asked for new points of discussion as well as their reactions to what previous groups had said. By the time the groups had rotated through all three rooms, no new discussion points were raised. We therefore assumed saturation of issues was reached. The same program was used the first evening; the two parents formed one group. The third PAP session was canceled since no parents attended. We did not have access to membership lists of the club but according to a board member, most of the parents of young talented gymnasts participated in one of the PAP sessions.

Due to COVID-19 lockdowns we were unable to hold this program at the second participating elite gymnastics club. We therefore held individual virtual interviews with eight parents (four mothers and four fathers) of this club. Again, similar informed consent procedures were followed. We used a semi-structured topic list, based on the program and outcomes of the elements raised in the focus groups of the previous evenings and asked the interviewees to expand on them if they so wished. They were also invited to raise new issues but they did not do so. This absence of new issues also suggests saturation of the elements comprising the three topics was reached. We recorded verbatim everything parents said in the focus groups and interviews and what was written on white boards. The data were subsequently transcribed word for word.

### Positionality

As three experienced professionals working in the field of physical education and sport at the university level, we acknowledge that our own biographies and our work in the area of pedagogy resonate in our research. Although all of the authors have an undergraduate degree in physical education, our only involvement in competitive gymnastics has been through a comprehensive research project focusing on elite gymnasts, their parents, coaches and the managers of the clubs of which they were a member. The current project is an extension of our this research endeavor in which we discovered that parents and club directors/managers were often blind to how elite gymnasts experienced their sport. During the current research project we therefore continually critically reflected on these assumptions to explore how they might have informed the ways in which we interpreted the data by using several iterations to reach agreement on our presentation of the results (Denzin and Lincoln, 2000; Boeije, 2010).

### Data Analysis

Analysis of focus group data situated within a poststructuralism perspective places little emphasis on “systematic analysis”, as groups are viewed as producing locally situated accounts (Freeman, 2006; Smithson, 2008). Our post structural analytic focus, therefore, was not on what individuals said in a group context but on how parents collectively negotiated and navigated discourses constructed within this group context about a “good” gymnast and being a “good” gymnastic parent.

Our analysis is situated in a post structural theoretical framework (Freeman, 2006; Smithson, 2008). We therefore do not assume linearity or exclusive coding/categorizations. The codes as well as the placement of the elements into a governmentality analytic overlap because parents are socialized into a complex assemblage. An assemblage assumes complexity and that therefore, its elements overlap.

Our analysis was based on procedures described by Boeije (2010). We used the group, rather than individuals as our unit of analysis. Our data analysis consisted of two major procedures: first, identifying components of the assemblage that reflected the issues raised by parents and second, detecting processes of governmentality that socialized parents into navigating these issues or elements in the desired “club” way. The focus of both analyses was on what was said or done rather than who said it. We therefore did not assign pseudonyms to parents, nor use any marker to describe them nor did we differentiate between parents of the two clubs.

Although the ways the focus groups operated resulted in a funneling of the data, we began the analysis anew using the analytical process proposed by Boeije (2010) for analyzing qualitative data. We began with open coding of the data to identify elements or issues that might be part of an assemblage. Each researcher did this independently after which we shared the resultant codes, i.e., elements. This process revealed that there were various disparate but interrelated elements that were part of being a parent of an elite gymnast at the clubs. Although individually we named the elements somewhat differently, we were in complete agreement in our identification of them. We, therefore, worked toward reaching unanimity in properly naming the issues or elements that had emerged in this initial analysis.

We subsequently, situated the elements in the literature and engaged in a selective coding process, that is, sorting/grouping the issues/elements and/or adjusting the name of an issue (Boeije, 2010). Again, each of the three researchers did this independently followed by an inter-researcher discussion in which we continually shifted between the data and our theoretical framework to identify key elements of an assemblage that disciplined parents and their daughters into dominant ways of thinking and doing at the clubs. For example, initially we agreed on the code “family” but after the selective coding process it became “disruptions of family constellations”. After discussions among researchers these issues were refined and selectively coded as: talent identification, (includes age), affect/joy (in skill mastery and in coach athlete relationships), hierarchical skill development plan, time spent including travel, family disruptions and forms of parental resistance. The socialization of parents was at the center of this complex assemblage. We subsequently used Dean’s (2010) dimensions of governmentality (technologies, rationalities, and culture/tradition) as an analytical tool to describe how parents were subtly governed into accepting the *status quo*. Specifically, we describe and analyze how participation was legitimized and enabled, how parents and their daughters were disciplined into docility through skill development plans and the coach-athlete relationship, how parents coped with the various issues that constituted the assemblage, how emotions and discourses about being a parent of an elite gymnast were entangled, and how parents resisted certain practices. Although these dimensions of governmentality and of affect were entangled with each other, we separated them heuristically to enable us to present the data. The resulting analysis revealed how participation was legitimized and enabled through talent identification, affect/joy and skill mastery; how parents and their daughters were disciplined into docility through hierarchical skill development, affect in the coach-athlete relationship and affect/joy. We expand on this assemblage in the “Results” section.

### Trustworthiness and Credibility

Denzin and Lincoln (2000) have argued that rather than focusing on absolute standards of reliability and validity associated with quantitative research, qualitative research analyses need to address trustworthiness and credibility. We believe that the parents were trustworthy in describing their experiences. Rather than using a list of topics we wanted them to discuss, we asked them to name and explain the issues they wished to discuss (Madriz, 2000; Freeman, 2006). The data, therefore, were parent-generated as parents presented their issues. Data saturation occurred as reflected in our use of progressive focus groups, in the absence of new issues emerging in the final round of the focus groups and in the confirmation of the issues by the interviewees who also did not raise any new issues. This process could be seen as a form of triangulation of method and preliminary analysis and speaks to the trustworthiness of both the data and the analytic process.

We strengthened the trustworthiness of our analysis by engaging in separate (critical) readings of the data and the subsequent steps of the analysis (Boeije, 2010). These separate readings led to discussions among the researchers. After each discussion we returned to the data and situated it in the theoretical framework. The analysis itself was a process of creating (and dismissing) critical interpretations. We assume, based on the foregoing, that our data are trustworthy and credible in reflecting how these parents experienced their daughter’s involvement in elite gymnastics. We do not pretend that we have uncovered THE truth about parents being socialized into gymnastics culture. Instead, we assume we have uncovered partial and situated truths (situated knowledge) about this socialization as described by these parents (Haraway, 1988).

## Results

**FIGURE 1:**
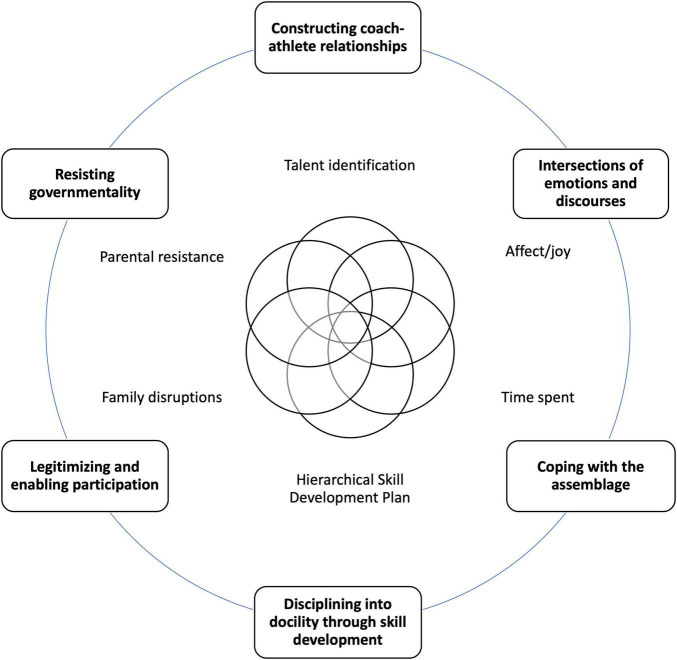
Assemblage of the socialization of parents.

The results suggested that although these parents problematized certain practices during PAP sessions, they continued to accept the regulations and norms created by the staff. This acceptance was a gradual process that occurred after an athlete was marked as having potential or talent and selected to be part of the elite club. This process involved an assemblage (see Figure 1) that drew parents into a web of compliance shaped by an entanglement of emotions, rationalities, the historical structure of technologies used to develop skill (the long term development plan), their daughter’s progress and interactions with other parents and coaching staff. Although we highlight the role of emotions separately, we note that affect was also entangled in all of the dimensions of governmentality. We briefly discuss each dimension of governmentality as we present it. In the general discussion we look at the findings using more of a helicopter view.

### Legitimizing and Enabling Participation

Young girls became involved in the clubs when they were identified as being gifted or talented and having potential. Both the staff and parents noted that their daughters early on displayed qualities assumed to be needed for high level gymnastics. Parents described how their daughters were physically energetic when very young and were constantly trying gymnastics stunts in and around the house:

“When she was small, she was always physically active. She would often do handstands in the living room”.

“As a young girl she would stand on her hands and watch tv upside down”.

“She could hang from a horizontal bar for a long time”.

These parents, therefore, looked for a gymnastics club where their daughter could be involved. When these girls joined a club, they were quickly identified as being gifted and as possessing potential to be outstanding talented gymnasts. Parents gave examples of how this worked:

An affiliate of the club for elite gymnasts began near our house; our daughter joined it when she was 4 years old; now she is 11. A few months after she joined the club she was already identified as being talented; she moved to the elite level when she was 5 1/2 years old.

Another described how gymnastics was supposed to be a stopgap measure during the winter field hockey stop:

She [our daughter] was involved in field hockey but had nothing to do in the winter. So, we checked out a nearby gymnastics club. Two weeks after she started there, she was already moved to the group identified as having “potential”.

Often local clubs had insufficient resources or possibilities for these girls to develop further. Parents then looked for elite clubs in other cities although going there meant travel by bike or train and/or parental chauffeuring duties. A parent explained what happened when their daughter joined a local club:

The highest level the local gymnastics club offered was the 2nd division. Few girls practiced at that level; she immediately won all the competitions. She was dissatisfied with only six practice hours per week and wanted more of a challenge. The local club could not offer her more. We then looked at the elite club in City X to see if that could provide her with the needed challenge and higher skill levels.

This search for an adequate club, instead of changing to another sport that could be done locally, was a crucial step in the entanglement of parents and their daughter with gymnastics, its culture and skill development model.

This identification of very young talented children for sport is part of the elite sport program of the Netherlands (Reijgersberg et al., 2010). This label of having “potential” in gymnastics has consequences, however, for the young girls and for their entire families. A parent acknowledged that “gymnastics seems to be a sport in which the more you practice, the better you become. It is not just about talent but also about practicing, much more than in other sports”.

This time element is considered to be a crucial part of becoming and being a good gymnast and disrupted regular family arrangements and routines. The following accounts by two parents revealed how talent identification can serve as a rationale for allowing a girl’s involvement in gymnastics to shape family arrangements:

I have three children. One wanted to play rugby. I told him he couldn’t because it asked too much: practices twice a week with competitions in the weekend. But then my daughter began gymnastics and she was very talented so we let her get involved. My son did not like that at first but he came to accept it.

We talk about this constantly. It [her participation] affects everyone in our family; we have to bring and pick her up since she is only 7. I cannot leave the youngest home alone so she has to come along. She (the youngest) always says “I’m doing bringing and picking up” as if that is her activity. How long can we continue to facilitate this?

Participation is not an individual matter but involves the whole family and their routines. A parent described how family routines have been adjusted to fit the talented daughter’s schedule: “There are also matters such as eating and going to bed on time. We have to tell the others they have to get going because their sister has to go to bed. She needs a lot of sleep”. Another parent conceded the need for changes in family routines for the sake of a talented child: “We struggle with the same thing. Can we ask everyone in my family to make sacrifices for one member?”

Family arrangements place the girl’s practice schedule at the center because that schedule is extensive and intensive. This rationality about elite gymnastics being a time-consuming sport gradually became an accepted regime of truth for these parents. A parent explained how they had to accept a shift from the original few hours per week of practice to many: “At first we said she could not practice so many hours in a week and not more than 4 days a week. Currently she trains 6 days per week and on some days has double sessions”. These disruptions were complex because involvement was linked to enjoyment:

The enjoyment of the other family members should not be sacrificed for the enjoyable experiences of one child. But I see how happy she is and how she loves it and that we can fit her schedule into ours. Then she does extremely well, wins a contest and comes home extremely happy. Can you deny her that?

A parent commented: “I see that she loves it, that she can continue to improve. The fact that you are one of the best in the Netherlands does a lot for a person”.

This compliance involving a gradual shift to many hours and the acceptance of that by parents and gymnasts, is an example of the subtle working of governmentality. These accounts about talent identification echo Kilger and Börjesson’s (2015) assertion that talent selection should be understood as a discursive repertoire embedded in a legitimacy bounded by what has been constructed as the identification of essential skills. Further on, we reveal how the staff used technologies to counter resistance to the time demands and how the resistance is diminished and absorbed.

### Disciplining Into Docility Through Skill Development

Once these girls became part of a club, they were embedded in its long term development plan based on a regime of truth how young talented gymnasts should be developed, the necessary skill progressions and what was needed to make them outstanding. In this manner, they and their parents were regulated into docility *via* normalizing judgments about talent development (Foucault, 1974).

As we explained earlier, the girls were part of a multi-level program. Each level consisted of hierarchical skill progressions and development. If an athlete was unable to practice for a while she might be dropped to a lower level. These levels and the differences between them formed a technology that pushed the girls to desire to be at the highest level possible. The negative consequences associated with possibility of going “down” a level meant it was difficult for parents to insist on a time out for their daughters or to protest the number of hours their daughter spent on gymnastics.

Parents were not happy with the time element as the data above revealed but as the fragments show below, they gradually were governed into accepting this regime as necessary. Although they problematized these practices, they were subtly led to comply with them. The ways in which the development plan was regulated and its entanglement with emotions, especially enjoyment, made it difficult for parents to pull their daughter out of gymnastics. A parent explained how the time involvement slowly increased making it difficult to limit involvement:

It went gradually. First the question was if she could also train Wednesday afternoons? Then later another afternoon was added. We thought: “OK she really wants this”. She came home very enthusiastic about her practice sessions. Then a fourth afternoon was added, which she really wanted. What is the limit of how much we let her be involved? That is difficult, who determines this limit? You really need the hours of practice but is it worth it? She has talent. Her limit is 32 h since she needs time to eat, go to school, etc.

The challenge and enjoyment the girls experience in skill mastery also contributed to the compliance of parents with the development plan. Two parents explained what skill mastery meant for their daughters and the emotions that accompanied working toward skill mastery: “My daughter loves the challenge of learning new skills. And then she practices, practices and practices and finally she is able to do it and thinks ‘Wow!”’ and: “My daughter is not busy trying to reach the top but mastering new skills”. This love of mastery also explains why dropping to a lower level was associated with less enjoyment. The following fragment reveals how the technology of a skill development plan ensured the girls did not resist the rules for skill development. A parent described how the coaches seemed to listen to the parents and girls about the number of hours of practice and gave them a voice in limiting the number of hours. In a short time, however, the girls ended up practicing the expected number of hours.

We told the coaches we thought the time required for gymnastics was becoming too much. They said she could cut back but then she would have to return to the non-elite level. They added that it was a shame to waste so much talent. They then let the girls decide how many hours they wanted to practice and told them they could remain part of the elite division even with fewer hours but that it was expected that over time, they would practice more hours. When our daughter heard she could decide herself and stay in the elite program, she said she would stay. In about 4 weeks she increased the number of hours so that she was practicing according to the expected norm.

Not only the ways vacation time is filled changed when a girl is marked as talented, but the training regime also shaped school plans. The parents attributed their acceptance of these changes to the daughter’s choices:

She used to attend advanced classes at the high school level. Now she has decided to stop that and devote those mornings to gymnastic practice instead. We and the school have discussed the consequences of this decision with her. She then was allowed to choose what she wanted to do we accepted her choice.

Other parents also constructed their acceptance of the gymnastics regime based on the choice their daughter made:

When you decide to be involved in elite sport then you have to sacrifice some things. We explained that to her and told her it was her choice. We did not push her to stay in the sport, we only tried to facilitate her choices.

This theme of choice is embedded in many rationalities parents used to justify their daughter’s participation. This emphasis on choice that is entangled with enjoyment reveals why it might be difficult for parents to safeguard the interests of their daughter.

The long term development plan is part of the assemblage that regulates processes of governmentality employed by the club. Parents take on narratives used by the coaching staff that injuries are par for the course and that if a girl drops out for a time, then she will never catch up. This element of the assemblage ensured the girls stayed in the program and did not take a time out for injuries because they would “fall behind”. A parent described how this played out:

There never is time for a break; yet the club says they take into account what this asks of the gymnasts’ bodies. Our daughter has problems with her foot regardless of the solutions they devise for her. She does not dare to tell the coach about this although the staff says they should be told of such things. So, then we choose and we keep her at home and let her miss a practice.

We note that the parents did not keep her at home until she is pain free or injury free. A parent explained how the enormity of hours of gymnastic practice shaped their daughter’s “choice”

If she wants to stop for a while, even if it is only half a year, then that is the end of it for her. If you become a gymnast at age 6 and you did if for 4 years and invested so much then it is difficult for a child to choose another activity.

The long term development regime used by the gymnastic clubs is both linear and hierarchical. This linearity is similar to the results found by Dowling et al. (2020) in their analysis of the Long Term Athletic Development plan used in Canada. The linearity and hierarchy of such long term development plans shape the structure of clubs because it consists of various levels. Gymnasts cannot fall behind. The gymnasts’ fear of dropping to a lower level, disciplined parents into accepting the structure. This Long Term Development plan for elite gymnastics in the Netherlands was controlled by the staff, primarily the head coaches. Parents and gymnasts had little to say in it. It was constructed as a regime of truth that was assumed to produce an “elite” athlete. None of the parents problematized the plan although they questioned the number of hours of weekly involvement it required. They accepted those hours as being a necessary part of the development of their talented daughter.

### Coping With the Assemblage

Parents are also asked to make sacrifices for the sake of their daughter’s development as a gymnast. There is literally little time off. A gymnast’s daily, monthly, and yearly schedule is shaped by the demands of the long term development program in which there is little room for accommodation of individual desires. A parent described the dilemmas surrounding family vacations:

Our family ski trips were an established ritual. At first our daughter wanted to continue to go with us but gradually she accepted that she could not go. We did not like it because our family consists of five members and not four.

Although parents problematized these disruptions, they were unable achieve changes to the schedule and its demands nor did they pull their daughter out of the club. They engaged in self-regulation to make the daughter’s schedule a reality and a necessity.

Parents also described how the staff subtly worked to achieve acceptance of the practice regimes:

We cut back the number of practice hours after we had moved and after a family member died. The coaches were unhappy about this reduction. They said it was OK but their attitude conveyed something else. I thought: “they do not want to hear that this [reducing the hours] is necessary or they do not understand why this is necessary”.

This unhappiness served as a way to govern parents and daughter so that these parents felt pressured to increase the number of practice hours as quickly as possible.

Parents had other concerns as well. They problematized the possible effect that the many hours of practice and the types of activities might have on their daughter’s body. Infertility was a major concern. A parent shared what they found on internet: “I googled a bit and then read that muscle mass should not be too large because that [large muscle mass] leads to a low percentage of body fat which then means you are infertile”. Another echoed this fear but took it a step further: “I am troubled by the idea that if you practice elite sport for a long time, that leads to difficulties in having children. I do not want this [her possible infertility] on my conscience”. Another problematized the exercises the girls needed do to be able to execute a full split: “When I see how much those girls are stretched then I am not sure how safe it is for them”. These parents have seemingly taken on a discourse about the frailty of girls’ bodies and the purported detrimental effect elite sport can have on their bodies. This biopolitical stance has been part of the (negative) discourse about the effects of girls and women engaging in competitive sports since the 1920s (Jackson and Tinkler, 2007). The clubs addressed this concern during an evening for parents during which a former athlete and coach challenged these myths. Interestingly, parents were not so much concerned about the possibility of their daughters being underweight and/or possibly developing eating disorders than they were about their fertility.

Despite attempts by the staff to counter parental rationalities with technologies such as member meetings, parents at times questioned if their daughter’s involvement was worth it. They did so by drawing primarily on neo-liberal discourses of utility and weighing that against her enjoyment. Ultimately, they all used affect, the enjoyment factor, to justify their daughter’s involvement. A parent admitted:

My daughter is not that good but she trains many hours per week. I then wonder: “Is this worth it?” She cannot use her skills later for a job or to earn money with it. She participates in it because it is fun and it increases her self-confidence and to perform under pressure. But you can achieve that in other ways as well.

Another had similar questions about her daughter’s participation after watching a documentary about mental and physical abuse in gymnastics:

The only thing my daughter knows is that gymnastics is lots of fun and is enjoyable. But there is so much more to do outside of gymnastics. She is only 6. When can we let her choose and when do we choose for her? I find that very difficult.

The emotions that accompany winning, governed the parents into complying with the program:

But it is difficult to set a limit as to how far you can go. She wants to win competitions and after that become even better and win more competitions at a higher level. How can you then say: “You have to stop?”

Although the foregoing describes the objections parents had to many parts of the training rituals at the club, they based their acceptance of these arrangements on their perceptions of their child’s enjoyment.

### Intersections of Emotions and Discourses

Parents claimed they used their daughter’s emotions as a guide for safeguarding her interests and wellbeing. Enjoyment was often the criterion for continuing in the sport and with the rigid training regime. Although identification of talent was the major reason these girls became involved in the first place, enjoyment was the central reason given by parents for permitting their daughters to continue participation “Gymnastics is primarily about enjoyment and having fun”. And: “If a child enjoys gymnastics a lot, who are you to say: it has to stop?”

Parents and daughters invested a great deal of affect/emotions in the sport and in their daughters. According to these parents’ success produced happiness. They argue:

“As long as she enjoys it, it is OK”,

“I see she is involved because she fully enjoys it. She does not feel pressure to perform, which is probably why she does so well”.

“I am pleased with her involvement and will continue to support her in this as long as she enjoys it and injuries can be contained”.

This emphasis on pleasure seemed to serve as the guiding principle for parents for allowing their daughter to engage with gymnastics, regardless of coaching methods and time involvement. Parents enjoyed watching their daughters flourish due to their success. They were pleased with her progress. They were proud to have a highly skilled daughter. Parents said: “It is special to have a daughter who is involved in elite gymnastics” and “Now that she has been selected to the Junior national team, she realizes she is more capable than she realized. She realizes she might be able to reach the finals at the national competition”. They also acknowledged that this elite level asks a lot: “If you do not work hard, you are out”. The emotions of parents also contributed to accepting the current arrangements or technologies “I love seeing how she develops as a gymnast and how much pleasure she gets out of being able to execute a skill that she was unable to do previously”. And: “No we do not feel we pressure her to win. She loves competitions. We just have to make sure WE stay calm”.

These perceptions and constructions of pleasure/fun and competence were the norm for continued participation. Ahmed (2010), in her discussion of the functions of promises of enjoyment or happiness, has argued that this promise has a governing character because it pushes individuals toward that that might bring that happiness. This promise played a large role in governing both parents and their daughters in their acceptance of practices that constituted the development of these gymnasts. Happiness also played a role in how parents regarded and accepted the behaviors and practices of the coaches of their daughters but possibly also contributed to parental inability to safeguard her interests and/or wellbeing and/or to mitigate the coach-athlete relationship.

### Constructing Coach-Athlete Relationships

Parents attached a great deal of importance to the athlete-coach relationship but also recognized there was little they could do to influence that. They did problematize some of the interactions between coach and athlete. They wanted the elite coaches to use positive pedagogical practices. “These coaches want the children to work very hard and trust them to do so. It is important that then they are complimented for their work”. Another recounted how a coach scolded a girl by saying: “Don’t think you will be allowed to compete if you work like this”. Several parents problematized negative pedagogical practices used by the top-level coaches:

They are not very pedagogically minded and say stuff at times when they should not. They also did that when we temporarily cut back the hours our daughter practiced. I think it was totally inappropriate.

A parent contended that what makes her distrust a coach is favoritism while another pointed out that remarks by a head coach can demoralize gymnasts: “The only thing that demotivates or discourages her [my daughter] are snide remarks by a coach”. Another parent complained:

The elite level coaches do not even write a summary of our discussion with them about our daughter’s development. Other coaches at lower levels do make such summaries. We talked about this with one of the assistant coaches, who understands but can do little.

The parents also did not like the negative attitude held by top coaches about schooling. “They have said that school is not that important”. Such an attitude by coaches suggests they see these gymnasts as objects with bodies to be trained (Donnelly, 1993). They do not see them as (little) girls who have been largely shut out of the usual life for girls their age.

Some of the parents expected their daughter to negotiate obstacles she may encounter in her relationship with a coach. “We push our daughter to address problems she has with the coach or coaching practices; we discuss it at home and then she talks it over with the coach. We try to stay out of it”. This “solution” seems to make the daughter solely responsible for her relationship with a coach and treats that relationship in a rational manner (Pike and Scott, 2014). Given the age of the gymnasts, the hierarchy that exists in elite sport and the authority assigned to coaches in the coach athlete relationship, this may not be the best way to safeguard a child. Other parents acknowledged that what a coach says influences young gymnasts but that they have little influence as parents because they are in a lower hierarchical position. For example, a parent was unhappy with some of the things the coaches said to the gymnasts. “I do not know if the coaches can change. They need to see the effect of their words. I can support my daughter but they have a larger influence. She is powerless with respect to them”.

The parents constructed coaches that treat gymnasts in a negative manner, as an exception, however. According to them, “Most coaches are not like that; they push hard but in a positive manner”. Parents seemed to accept the current coaching situation because they had experiences with other coaches at lower levels who used positive pedagogical practices. None of the “other” coaches are elite head coaches, however. Other studies of athlete-coach relationships suggest that a head coach is given a great deal of room to engage in punitive practices because of his or her perceived knowledge and expertise (Kerr and Stirling, 2012; Jacobs et al., 2017; Smits et al., 2017; McMahon et al., 2018). Parents who were part of PAP sessions wanted to learn how to deal with, to ignore or to address such practices. Learning how to work with or around this behavior may, however, suggest acceptance. An angry parent objected to having to learn to deal with specific coaching behaviors that she/he thought were inappropriate. S/he exclaimed: “I do not need to be taught how to deal with that behavior since such behavior is simply wrong!” These parents problematized coaching behaviors and tried to bring about change by talking to assistant coaches.

There were few reports of parents confronting the elite coaches directly. Parents attributed a great deal of authority to head coaches. By doing so, they were disciplined into tolerating coaching behaviors and working around them. This way of working served as a technology that enabled coaches to ignore how the lives of these gymnasts and their complex daily realities may be at odds with the “real” world in which they live. They are both children and gymnasts but the child part faded into the background possibly because it was not embedded into the structured development plan of the clubs that focused solely on developing talent. The process of governmentality ensured that parents in general came to accept this (Bailey and Collins, 2013; Dowling et al., 2020). Although they did not agree with the nature of the interactions of some of the coaches with their daughters, parents normalized the behavior. Similar to those involved in the McMahon et al. (2018) study cited earlier, these parents seemed to assume that punitive coaching behaviors are a normal part of gymnastic culture. A parent asserted that: “I’ve read the media accounts and the [critical] book by retired gymnasts, but I do not see that type of behavior as mentioned in that book”. This normalization suggests processes of governmentality have worked well to ensure these parents accepted behaviors as normal while also acknowledging that these behaviors may not be pedagogically acceptable.

Parents were inclined to acquiesce to the ways coaches behaved and to the development plan because they had few notions of what elite gymnastics demands. They realized they had a knowledge and experience deficit concerning the practices of elite gymnastics. “The coaches simply want them to work very hard. They do not ask too much of her. This is what elite sport is and we tell her that continually”. A parent described how their own sport history is quite different:

We [parents] have played another sport competitively. Things were quite different there. We have to trust these [gymnastic] coaches including knowing what “normal” growth and development is and that the injuries are part of this development.

And:

Her ambition is to go to the Olympics. I think: “Why not? Hold on to your dreams and why shouldn’t you reach that level?” I also know the road to achievement is long and difficult. Everything is possible if you work hard, make a commitment to your goals, have a bit of luck and stay injury free.

This acknowledgment of their deficit in knowledge and expertise and their acceptance of coaching behaviors they do not like, therefore, served as a technology that limited parents in their ability to safeguard their daughter’s interests. Discursive power is never complete, however. There is always room for resistance (Foucault, 1974, 1991).

### Resisting Governmentality

The foregoing revealed how the clubs navigated the resistance of parents of arrangements that seemed problematic by introducing a measure gradually. Problematization was not just confined to head coach behaviors but included other issues such as communication between the club and parents. The clubs attempted to communicate with parents and children through Instagram, Facebook and an irregular newsletter and a member council. A parent agreed that “Communication with the coaching staff is not ideal yet but getting better”. Another parent, however, contended that communication by the coaching staff with parents and gymnasts was ineffective/lacking. For example, a parent contended that “coaches want the girls to be independent. But then they tell these girls so much in a meeting, that the girls forget. A week later my daughter says something that she remembers from the discussion”.

Parents were able to push for change on several issues with respect to physical and mental wellbeing of their daughters. At first, only those gymnasts who were part of the top level could make use of the physiotherapist and the mental coach. Parents who wanted access to these professional services initially had to pay for this themselves. The only exception to this rule was that the club paid for these services for those who competed at the international level. The parents argued that all children should be able to access this support. “There are fewer support resources for parents of pupils and juniors than for seniors. We don’t agree because all those girls train an equal number of hours!” These complaints from parents have had some effect: The services of the support staff have been gradually changing to encompass all the children. Thus parents were able to transform several of their problematizations of technologies into structural change. Such transformations were rare.

In summary then, the parents of elite young gymnasts were governed into acceptance of an assemblage of dominant club practices infused with emotions such as pleasure and enjoyment. This assemblage included the identification of their daughter as being talented and as enjoying her development as a gymnast, an almost unquestioned adherence to a hierarchical skill structure, colonization of time by the staff that resulted in many hours of practice and disruptions of family constellations, affect in coach-athlete relationships and moments of parental resistance. The socialization of parents is at the center of this complex assemblage.

## Discussion

The data analysis revealed that parenting of these elite gymnasts occurred in a highly affectively charged space; both parents and gymnasts had a large emotional investment in their participation in the programs. The successful development of the gymnasts produced happiness; it was that happiness that enabled both parents and gymnasts to continue to be involved even when they did not agree with club policies or behaviors of some of the coaches. Over time, parental voices became compatible with the objectives of the coaching staff due to the technologies employed by the staff. For example, the staff listened and did not say no to the parents’ request to reduce the number of training hours but instituted a process that gave parents and gymnasts a say in the number of weekly hours spent training. Within 4 weeks the girls had returned to their usual training routines. This entanglement of emotions with rationalities used by parents to allow their daughter’s involvement to take up most of her time and disrupt the whole family suggests why making parents primarily responsible for safeguarding their daughter’s wellbeing in elite gymnastics may be inappropriate. The emotions and the construction of their daughter as talented combined to produce compliance even when parents problematized certain practices. These problematizations were, however, largely based on rational thought, rarely produced alternative discourses and rarely brought about significant transformations of technologies and rationalities that comprised elite gymnastics at these clubs.

The regulatory processes of these clubs based on the skill development framework normalized certain standards of behavior for parents and gymnasts. For example, the many hours of practice, rewards, exclusion or inclusion from certain groups and the learning/progressions that promoted skill mastery, instilled in parents and their daughters club norms and the desire to comply with these norms.

The governmental process that socialized these parents into being good gymnastic parents may have been strengthened by the societal discourse of what it means to be a good parent when a child is involved in sport. Coakley (2006) has argued that many parents believe that organized youth sport is a valued site where children build character, learn to cooperate and compete and to take on responsibility. Trussell and Shaw (2012) found that parents of young athletes were actively involved in their child’s participation and allowed it to disrupt family rituals and resources because they felt “their moral worth as a parent [was] evaluated by their children’s successful participation in youth sport, and the parents’ visible investment in this pursuit.” (p. 390). Consequently, these gymnastic parents may have been governed in various ways to accept the norms of conduct in these gymnastic clubs.

When parents and their daughters as well as the coaching staff assumed these norms were common sense, they were exercising power over themselves (Gallagher, 2008). These norms were coercive but also became productive. The girls mastered skills, did well in competitions and were able to move through skill progressions to learn even more challenging skills. This mastery produced enjoyment upon which parents based their decision to allow her to continue. This productive nature of governmentality strengthened the idea that the practices and other elements of the assemblage that produced club culture were normal and common sense. Whatever role these elements comprising this assemblage actually played in this normalization and acquiescence, up to a point they possessed their own:

specific regularities, logic, strategy, self-evidence and “reason”. It is a question of analyzing a “regime of practices” – practices being understood here as places where what is said and what is done, rules imposed and reasons given, the planned and the taken for granted meet and interconnect (Foucault, 1991: 75).

The results revealed that processes of governmentality resulted in these parents moving into the acceptance/acquiescence stage of what Kerr and Stirling (2012) have called consecutive steps of grooming by coaches and staff that can normalize abusive practices. The first step consists of talent identification followed by parents being asked to trust the coach (step 2) and thus relinquishing a great deal of control over their daughter’s wellbeing. The data revealed how these steps of talent identification and being disciplined into obeying/trusting the coaches disciplined the parents and gymnasts into self-regulation. The parents had concerns, however (step 3) and used PAP to problematize certain practices such as time spent, demotions through levels, disrupted family vacations, and negative pedagogical practices by coaches. However, the data analysis suggested parents were subjected to processes of governmentality by the staff that constructed these practices as normal. In this manner the complicity of parents and their daughters (as well as the staff) were continuously secured (step 4) despite parental resistance. These young gymnasts, therefore, will continue to be vulnerable to abusive practices during their gymnastic careers since their parents may be able to do little to safeguard their wellbeing, unless she stops her involvement in gymnastics. Step 5 of Kerr and Stirling’s model of progressive steps of grooming pertains to coaches engaging in emotional, sexual and/or physical abusive behavior often with little resistance or knowledge of its occurrence by parents. Lang (2010a) has argued that the number of hours these girls spend on the sport can be seen as abusive. She contended that:

recognition that such long training hours have become normalized as part of the discursive regime of elite youth sport has led to suggestions that elite youth sport more closely resembles the adult world of work than the child’s world of play, and that child athletes are being exploited in ways that would not be tolerated in other social settings (p. 60).

The gradual aspect of this process of governmentality that normalized these practices as well as the many issues that comprised its assemblage make it extremely difficult to hold these parents solely responsible for safeguarding the well-being and interest of their daughters in elite gymnastics. The technologies of the development plan with its various hierarchical levels, the purported enjoyment of the girls and the authority granted to coaching expertise and experience exercised power that socialized parents into acceptance and acquiescence of the assemblage that constituted elite gymnastics for young girls.

The responsibility for safeguarding described at the beginning of this manuscript seems to assume parents are not absorbed into the organizational and practice culture of a sport club and that they are able to be critical of and resist club norms and coaching practices. The results suggest this assumption is unfounded and not situated in the complex assemblage shaping gymnastic practices. This emphasis on individuals shaping their own subjectivities is significant, for it extends the terrain of government even further into the very depths of the soul (Rose, 2000).

## Conclusion

The results revealed that being a parent of an elite gymnast who has been identified as having talent is comprised of complexities that form an assemblage. This assemblage consists of issues such as parental acceptance of discourses about the development of an elite gymnast, parental involvement based on perceptions of their daughter’s enjoyment of skill mastery, the authority parents granted to coaches and expertise, the required flexibility of family structures and of other societal structures such as schooling. These intersected and produced the “good” gymnast and the acquiescent parent. Young gymnasts and their parents were socialized by these elements into what seems like total colonization of time and energy to adhere to the rationalities or discourses about development of young talents and what it means to be an elite gymnast and her parents.

### Limitations and Future Directions

This study foregrounded relations of power exercised through governmentalities that were constituted in an assemblage of various elements. The findings suggest that the use of the concepts of assemblage, discourses, affect and governmentality enabled us to go beyond descriptions of coach-athlete interactions and to uncover complexities of changing practices in elite WAG and of supporting parents who are unable to enact change. The results also revealed the necessity of taking the entanglement of emotions and discourses into account in the study of sport practice. We did not ascertain the extent to which these findings were specific to the performance culture in elite WAG. Possibly other sports may be constituted by similar assemblages at the elite levels, even if athletes may begin at a later age than do gymnasts.

Our research focus was on parents of the youngest gymnasts who had been identified as talented. The replication of this and earlier studies on WAG in various countries could reveal the extent to which the assemblages that comprise systems and coach training may vary by country. Such investigations may offer other insights for possible change. Further research also needs to explore under which conditions parents are able to resist processes of governmentality and how these processes may stimulate them to withdraw their daughter from elite WAG. Such knowledge may assist parents in devising strategies that enhance their ability to safeguard the wellbeing of their daughters in WAG.

## Data Availability Statement

The datasets presented in this article are not readily available because we promised confidentiality and privacy to the participants. Since the number of elite gymnasts in the Netherlands is small, the availability of the datasets would easily reveal the identities of the parents. Requests to access the datasets should be directed to the corresponding author.

## Ethics Statement

Ethical review and approval was not required for the study on human participants in accordance with the local legislation and institutional requirements. The patients/participants provided their written informed consent to participate in this study.

## Author Contributions

All authors listed have made a substantial, direct, and intellectual contribution to the work, and approved it for publication.

## Conflict of Interest

The authors declare that the research was conducted in the absence of any commercial or financial relationships that could be construed as a potential conflict of interest.

## Publisher’s Note

All claims expressed in this article are solely those of the authors and do not necessarily represent those of their affiliated organizations, or those of the publisher, the editors and the reviewers. Any product that may be evaluated in this article, or claim that may be made by its manufacturer, is not guaranteed or endorsed by the publisher.
